# Self-healing behavior of recycled asphalt prepared by residue oil of straw liquefaction based on molecular dynamics simulation

**DOI:** 10.1038/s41598-022-06743-1

**Published:** 2022-02-17

**Authors:** Xuewen Zheng, Wenyuan Xu, Kai Cao, Keke Li

**Affiliations:** 1 School of Civil Engineering, Northeast Forestry University, Harbin, 150040 Heilongjiang China; 2 College of Civil Engineering and Architecture, Zhejiang University, Hangzhou, 310058 Zhejiang China

**Keywords:** Biomaterials, Civil engineering

## Abstract

In order to accurately describe the self-healing behavior of recycled asphalt prepared by residue oil of straw liquefaction (ROSL), five ROSL contents of 2%, 4%, 6%, 10%, and 15% were added to the aged asphalt to represent the recycled asphalt and are denoted as ROSL-2, ROSL-4, ROSL-6, ROSL-10, and ROSL-15, respectively. Molecular simulation was used to simulate the healing behavior of nano cracks. A three-layer system was established in which the two sides are asphalt molecules and the middle is a 30 Å vacuum layer. The vacuum layer represented the internal nano cracks in the asphalt. The results show that the disappearance of cracks during the self-healing process was the result of the combined effect of model volume compression and asphalt molecule stretching. Self-healing is mainly affected by the van der Waals forces between the molecules. The self-healing rate of recycled asphalt is closely related to the content of ROSL, the higher the ROSL content, the greater the diffusion coefficient, which is more conducive to asphalt self-healing. However, as the time for ROSL-10 and ROSL-15 to reach the equilibrium distribution of relative concentration and density stability is basically the same, and the diffusion coefficient of ROSL-10 is basically the same as that of virgin asphalt, the optimal content of the ROSL is recommended to be 10%.

## Introduction

The reuse of waste asphalt pavement materials in recycled asphalt pavement (RAP) is not only beneficial for reducing the cost of pavement construction but also for reducing solid waste and saving raw materials. It is one of the most recycled materials worldwide^1^. Under the comprehensive influence of environment and load, the asphalt in the old pavement is oxidized and aged, causing a change in the asphalt components, such as a volatilization of light components and increase in the concentration of polar molecules (such as asphaltene), and an increase in polar molecules easily causes agglomeration^2^. Compared to virgin asphalt, RAP binder is oxidized and hardened during service period. Thus, the blending of virgin asphalt with RAP binder results in the stiffer binder than virgin asphalt and several concerns, such as cracking, peeling, and poor durability^3^. Adding rejuvenator to aged asphalt is an effective means of solving these problems by making the RAP asphalt binder effectively useful for blending with virgin asphalt, reducing the RAP asphalt binder stiffness. Research has shown that rejuvenators can restore the properties of aged asphalt and meet the requirements as virgin asphalt^4^.

Based on the source of raw materials, the rejuvenator can be divided into two categories: petroleum-based and bio-based^5^; however, considering economic and environmental factors, bio-based rejuvenators have attracted more attention. For example, the bio-oil extracted from waste cooking oil can soften aged asphalt; it has good compatibility with asphalt under static storage conditions and improves the low-temperature performance and construction workability, and compensates for the light components of aged asphalt, and relieves the polymerization of polar components^6,7^. Compound rejuvenators made from high-protein algae and high-fat animal manure can not only restore the molecular conformation of asphalt but also restore physical, chemical, and rheological properties^2^. Moreover, Oldham et al.’s research showed that bio-oil extracted from pig manure can improve anti-water damage property of asphalt^8^. Zadshir et al. reported three sources of rejuvenators, namely, petroleum-based, chemically modified vegetable oil, and bio-oil from pig manure. Their research indicated that the three rejuvenators successfully reduced the size ratio of macromolecules in the aged asphalt and balanced the colloidal structure of asphalt disturbed by aging, and the thermodynamic properties of aged asphalt were effectively restored^9^. Aromatic extraction and waste vegetable oil were used as rejuvenators in Yu et al.’s research. The results showed that the mechanical properties of recycled asphalt were between those of virgin asphalt and aged asphalt; however, the rejuvenators could not reconstruct the microstructure of the aged asphalt^10^. The rejuvenator produced from soybean oil can reduce the glass transition temperature of the aged asphalt binder and improve its low-temperature performance^11^. The bio-oil produced from waste wood can reduce the mixing temperature of the asphalt mixture and improve the high-temperature performance but sacrifices the medium- and low-temperature performances^12^. Recycled asphalt prepared by the residue oil of straw liquefaction (ROSL) has good performance indices and can effectively improve the low-temperature performance of virgin asphalt^13,14^. Dong et al. investigated the road performance of recycled asphalt mixtures prepared from corn residue and concluded that the recycled asphalt could partially replace petroleum-based asphalt^15^.

The above mentioned studies include the research on bio-oil as a rejuvenator. It can be seen that all bio-oil rejuvenators can improve the asphalt performance, and some researchers have explained the corresponding modification mechanism from a micro point of view. In recent years, asphalt self-healing properties have been the focus of research from the macroscopic, microscopic, and nanoscale perspectives. For example, using a dynamic shear rheometer, Bai et al. evaluated the influence of oil extracted from waste wood on the self-healing performance of recycled asphalt from the perspective of energy and chemical structure^16^. Hao et al. carried out fatigue healing and cyclic loading tests in a time scanning mode and analyzed the effects of temperature, time, aging degree, damage degree, rejuvenator type, and rejuvenator content on the self-healing performance and fatigue life of recycled asphalt^17^. At the microscale, Su et al. studied the self-healing mechanism of aged asphalt microencapsulated with a rejuvenator by combining environmental scanning electron microscopy (ESEM), thermogravimetric analysis (TGA), and fluorescence microscopy^18^. The authors then used scanning electron microscopy (SEM), transmission electron microscopy (TEM), and Fourier transform attenuated total reflectance infrared (FTIR-ART) to investigate the final state of the microcapsules in the asphalt binder after self-healing^19^. From nanoscale, atomic force microscopy (AFM) combined with the phase field method is an effective means to study the asphalt self-healing mechanism^20^. In addition, molecular dynamics (MD) simulation is a commonly used method at the nanoscale^21,22^. Many scholars have evaluated the self-healing ability of asphalt^23,24^, crack evolution during asphalt self-healing^25^, self-healing of asphalt with cohesive failure^26^, self-healing and interfacial properties of rubber-modified asphalt^27^, and self-healing characteristics of asphalt^28–30^, which covering all aspects of factors affecting self-healing. As can be seen from the above studies, MD as a nanoscale research method provides a new way to study asphalt self-healing behavior.

In summary, bio-oil from a variety of sources can be used as asphalt rejuvenators, and previous studies have confirmed that bio-oil has good modification performance. ROSL is a rejuvenator, and researchers have also conducted a preliminary exploration of its modification performance^13,14,31,32^. However, most of these studies on ROSL modified asphalt are limited to macro mechanical properties and micro properties, they ignored the self-healing behavior and mechanism of ROSL on recycled asphalt. Therefore, in order to explore the above problems, in this paper, the self-healing behavior of recycled asphalt prepared by ROSL is studied based on MD simulations. The molecular model of asphalt was established by MD and validated with the density, glass transition temperature and solubility parameters. Once the model is validated, an interface system was created by embedding a vacuum pad of 30 Å between 2 groups of asphalt models to study the evolution of the self-healing process. Aged asphalt and virgin asphalt were also studied to compare the components that determine the diffusion rate. To enable the easier understanding of the figures and descriptions, through the remainder of this paper, ROSL and rejuvenator refer to the residue oil of straw liquefaction.

## Research method

### Molecular structures in MD

**Figure 1:**
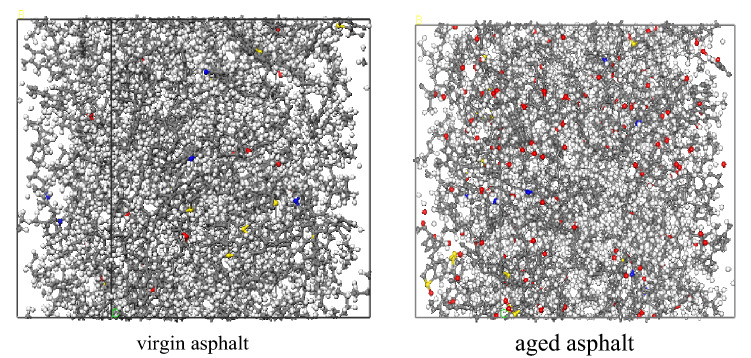
The virgin asphalt and aged asphalt MD models.

Asphalt is composed of a variety of compounds, and because of its complex structure, the separation of pure compound monomers is very difficult technically. Previous studies have shown that using an average molecular structure to represent real asphalt is an effective tool for studying the physical and chemical properties of asphalt^33,34^. In recent years, the asphalt model has evolved from 3-component and 4-component models to a 4-component, 12-molecule model; the main components of the 3-component model are asphaltene, aromatic, and saturate, and the asphaltene 1,2 structure is usually adopted in the model proposed by the literature^33,34^. Guo et al. used n-docosane (n-C_22_H_46_) to represent the molecular structure of saturate^35^, aromatics were characterized by 1,7-dimethylnaphthalene, and the molecular mass ratios of each component of the 3-component model were asphaltene:saturate:aromatic = 20:20:60^23^. The main components of the 4-component model are asphaltene, saturate, naphthene aromatic, and polar aromatic. Chu^36^ and Yu^37^ applied the 4-component model to study the interface interaction between the asphalt and aggregate and the interaction between asphalt molecules and water molecules, and achieved satisfactory results, which confirmed the effectiveness of the 4-component asphalt model. The 4-component, 12-molecule model is currently the most popular molecular model. It is constantly being improved on the basis of the 3- and 4-component models, and the component structures are more detailed. Li et al.^38,39^ proposed an improved 12-molecule asphalt model in which the proportion of different molecular numbers was adjusted to make it more consistent with the three representative asphalts in the Strategic Highway Research Program (SHRP): AAA-1, AAK-1, and AAM-1. The aged asphalt model considers the aging mechanism; ketones and sulfoxides are introduced to replace the sensitive functional groups that are easily oxidized in the virgin asphalt^40^, and Hu and Qu et al.^41,42^ considered the oxidation level of asphalt molecules and suggested adjusting the number of ketones and sulfoxides to represent asphalt molecular models at different oxidation levels. In this study, according to AAA-1, models of virgin asphalt and aged asphalt were built, and their plausibility has been previously verified by Cui et al.^41^ and Greenfield^38^. The molecular structure and ratio are shown in Tables 1 and 2, and the asphalt model is shown in Fig. 1. The principal components of ROSL have been chemically characterized with GC–MS (gas chromatography–mass spectrometry), and the principal components of ROSL are shown in Table 3. The concentration ratio of 2H-Pyran-2-one, tetrahydro-6-methyl-, 1,3-Dioxane, 4-methyl-, Propanoic acid, 2,2-dimethyl-, 2-Butene, 1,1-dimethoxy- and 5,6-Epoxyhexanol-1 is 1:1.28:1.63:1.18:2.82, and the molecular number ratio calculated according to this concentration is 1:1:2:1:3 respectively. Then calculating the mass of asphalt molecules according to the molecular number in Tables 1 and 2, and five ROSL contents of 2% (3 ROSL molecules), 4% (6 ROSL molecules), 6% (10 ROSL molecules), 10% (17 ROSL molecules), and 15% (27 ROSL molecules) were added to the aged asphalt to represent the recycled asphalt according to the percentage of asphalt molecular weight and are denoted as ROSL-2, ROSL-4, ROSL-6, ROSL-10, and ROSL-15, respectively.

**Table 1:**
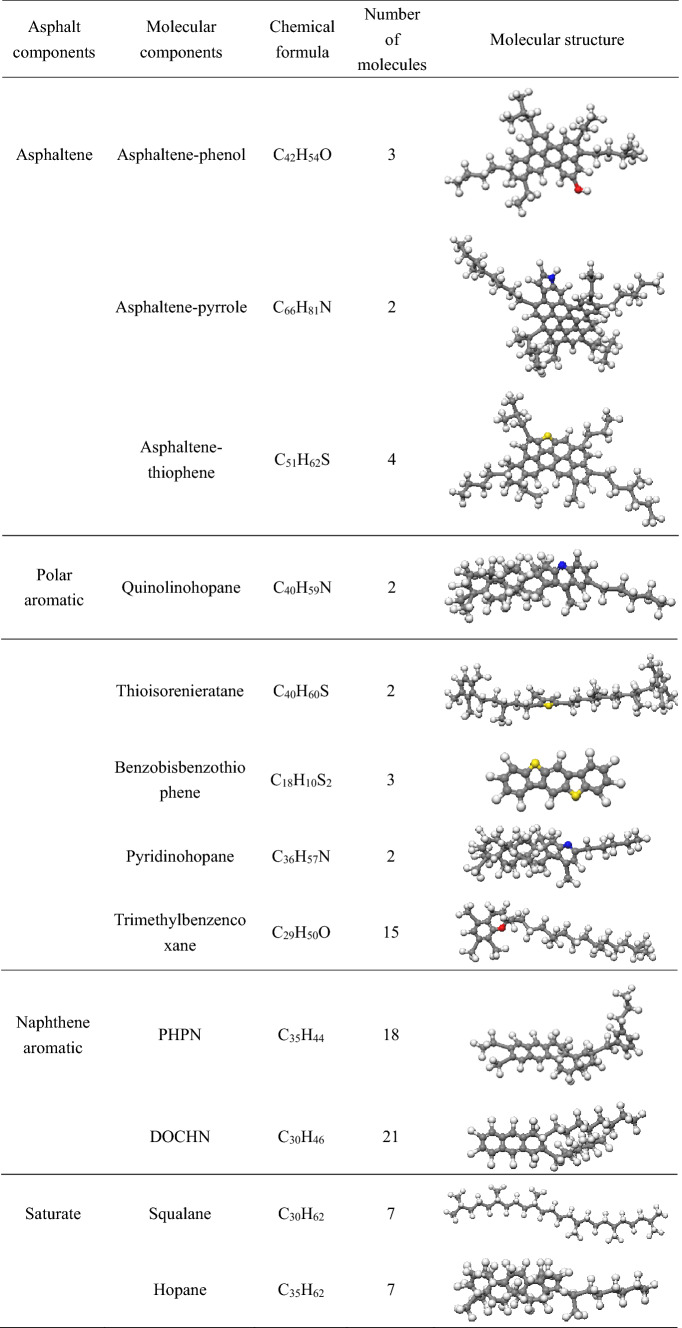
Molecular composition of the virgin asphalt.

**Table 2:**
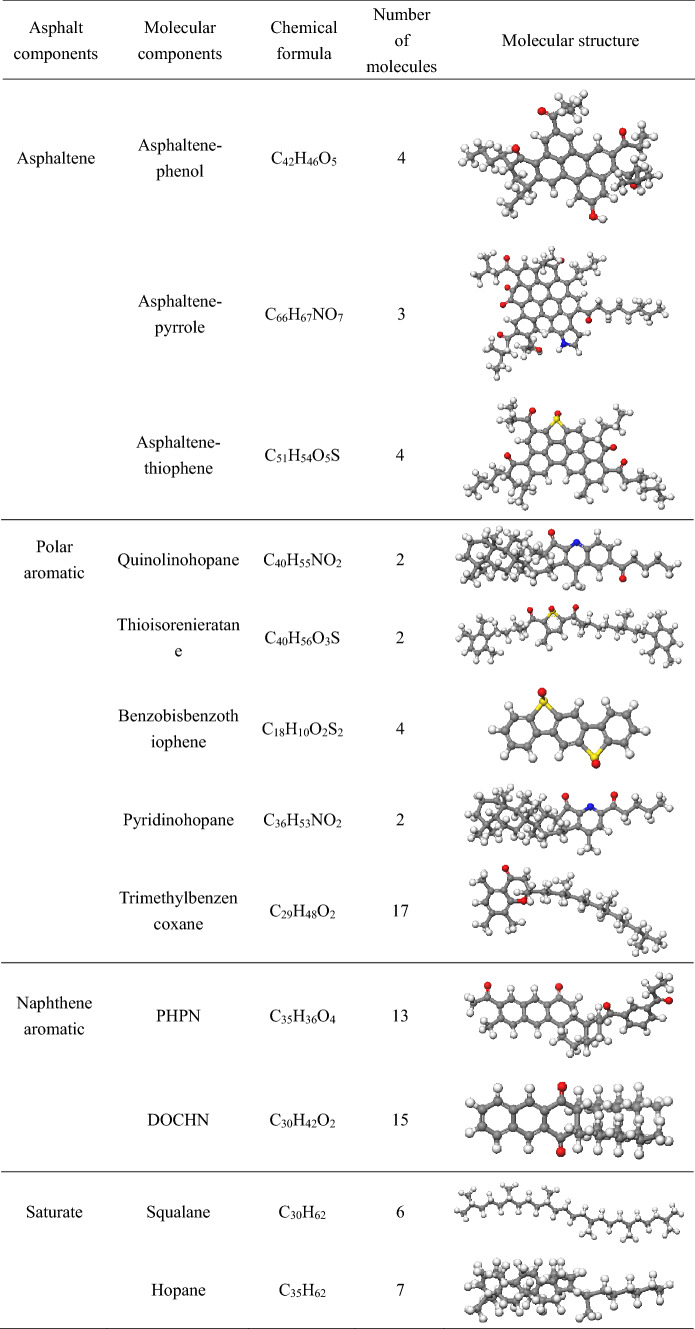
Molecular composition of the aged asphalt.

**Table 3:** The principal components of ROSL.

Components	Chemical formula	Area %
2H-Pyran-2-one, tetrahydro-6-methyl-	C_6_H_10_O_2_	13.657
1,3-Dioxane, 4-methyl-	C_5_H_10_O_2_	15.631
Propanoic acid, 2,2-dimethyl-	C_5_H_10_O_2_	19.956
2-Butene, 1,1-dimethoxy-	C_6_H_12_O_2_	16.358
5,6-Epoxyhexanol-1	C_6_H_12_O_2_	39.236

The simulation tool used in this study was Materials Studio. The COMPASS II (condensed-phase optimized molecular potentials for atomistic simulation studies) force field was selected to describe the interaction between molecules and the potential energy between molecules. The COMPASS II force field is suitable for the simulation of most common organic molecules, polymers, gas molecules, and some inorganic materials such as ordinary metals, metal oxides, metal halides, and zeolites and can accurately predict the properties of related materials^31,32^. The Nose–Hoover and Berendsen algorithms were selected to control the temperature and pressure, respectively. To accelerate the random combination of molecules in the asphalt system and make system reach equilibrium and reduce molecular chain entanglement, inspired by the long simulation time of previous researchers^25,43,44^, the following simulation steps were adopted in this study: (1) in the Amorphous Cell module, the lattice adopted periodic boundary conditions, the initial density was set to 0.1 g/cm^3^, and the asphalt system model was built according to the molecular proportion; (2) geometric optimization was performed to ensure that the initial energy was minimized; and (3) in the constant temperature and volume (NVT) ensemble, the temperature was set to 500 K, and the simulation step was 0.1 fs, then a 50 ps simulation was carried out for equilibrium of the system and reducing deviation in total energy between successive steps. (4) The simulation step of 1 fs was selected to further randomly move molecules in the NVT ensemble, the temperature was selected to be 500 K, and the total time was 300 ps. (5) In the isothermal-isobaric (NPT) ensemble, the molecules in the system were compressed, the pressure was set to 1 atm, the temperature was 500 K, the simulation step was 1 fs, and the total time was 500 ps. (6) Finally, a MD simulation of 2 ns was performed in the NVT ensemble to achieve a stable equilibrium state.

### Model validation

**Figure 2:**
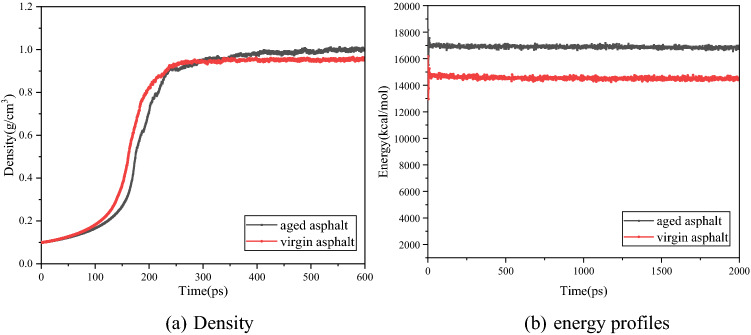
Simulation equilibrium: **(a)** density, and **(b)** energy profiles.

**Figure 3:**
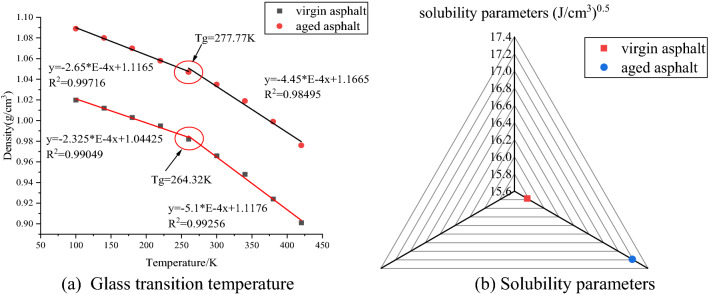
Glass transition temperature and solubility parameters of asphalt models.

To confirm the rationality of the asphalt models, the energy, density, glass transition temperature (T_g_) and solubility parameters of the system were selected as the verification parameters for the asphalt system. The simulation conditions were as follows: temperature was 298.15 K; time step was 1 fs; total simulation time was set to 600 ps in the NPT ensemble to stabilize the density, and a 2 ns simulation was performed in the NVT ensemble to ensure that the system reached complete equilibrium. The simulated density and energy of the asphalt system change with time as shown in Fig. 2. As shown in Fig. 2a, both the density and energy of the simulated system reach equilibrium and a stable state, and the equilibrium density of virgin asphalt was approximately 0.953 g/cm^3^, which is close to the true value of 1.01–1.04 g/cm^3^^45,46^ but slightly lower. There are two reasons for this phenomenon. Firstly, compared with the real asphalt system, only representative molecules are selected in the model. Because of the limited number of representative molecules, the density is slightly lower, which is similar to the simulation results of Xu^47^. In addition, the cut-off distance of the model is smaller than the model size, and the spatial scale of the model is larger than the cutoff distance; therefore, the molecular cohesion and model density are smaller^5^. The density of aged asphalt is approximately 1.008 g/cm^3^, which is higher than that of virgin asphalt and consistent with common knowledge. However, the density of the aged asphalt was also lower than the experimental value. In addition to the two reasons mentioned above, another reason may be that the model construction only considers the oxidation effect of asphalt molecules^48^. Moreover, it can be seen from Fig. 2b that the simulation time is sufficiently long and that the energy of the system reached equilibrium and a stable state. Furthermore, as can be seen in the Fig. 3, the calculated solubility parameters of virgin asphalt and aged asphalt were 15.773 (J/cm^3^)^0.5^ and 17.187 (J/cm^3^)^0.5^, respectively, and the T_g_ of virgin asphalt and aged asphalt were 264.32 K and 277.77 K, respectively, the which is consistent with the ranges found in previous studies^25,26,45,46^. It can be seen from Fig. 3 that the T_g_ and solubility parameters of asphalt increase after aging. From the perspective of molecular motion, the glass transition phenomenon indicates that the chain motion of the material begins to be frozen. The lower the T_g_, the better the low temperature performance of asphalt. The T_g_ of aged asphalt is higher than that of virgin asphalt, so the low temperature performance of aged asphalt becomes worse. The solubility parameter is closely related to the cohesive energy density. It is a measure of intermolecular force and reflects the aggregation state between molecules. The more molecules gather, the greater the cohesive energy and the greater the solubility parameter. The solubility parameter of aged asphalt is greater than that of virgin asphalt. Therefore, the molecular aggregation of aged asphalt is more severe. The reason for the solubility parameters and T_g_ is that aging reduces the light components and increases the asphaltene content. At the same time, the polarity of asphaltene after oxidation is enhanced and tends to aggregate, resulting in the enhancement of intermolecular gravity and the decline of chain activity. The simulation results show that the established asphalt system models are reasonable and can accurately predict the physical properties of the materials.

### Self-healing model of MD simulation

The fracture behavior of materials has a strong multiscale characteristic in nature, and different types of cracks can be observed in asphalt materials, ranging from nano/meso level to macro level. The crack width of nanoscale is around 10^-9^ m, and those of mesoscale crack and macroscale crack are around 10^–5^ m and > 10^–3^ m respectively. Besides, some researchers have showed chains can diffuse across the crack faces by wetting and potential barriers associated with inhomogeneities disappear. The long-range attractive interactions (electrostatic) between non-bonded segments at the interface are restored and thus drive adsorption, which defines the cohesive area with separation distances of about 0.3 nm to 10 nm. Short-range attractive interactions (acid–base or hydrogen bonds) are established at distances < 0.3 nm, and assure transfer of matter between adhering surfaces^49^. The behavior of healing occurs across a crack interface. This paper mainly studies the healing behavior of cracks on nanoscale, and MD is an important research method to study nanoscale crack healing^23,25^, thus, MD is selected to study the self-healing behavior of recycled asphalt. In order to determine the self-healing property of asphalt, an interface system was created by adding a vacuum pad 30 Å on one side of a unit amorphous cell, and a layer with artificial crack representing self-healing interface, and the crack width selected has been identified by previous studies^23,25,27,28,49,50^. After the asphalt and rejuvenator models for the molecular simulation were identified, a 30 Å vacuum layer was inserted between the models to represent microcracks inside the asphalt. Subsequently, each asphalt model was simulated in the NPT ensemble for 500 ps at 1 atm pressure, with a time step of 1 fs, and a conformation was output every 5000 steps.

**Figure 4:**
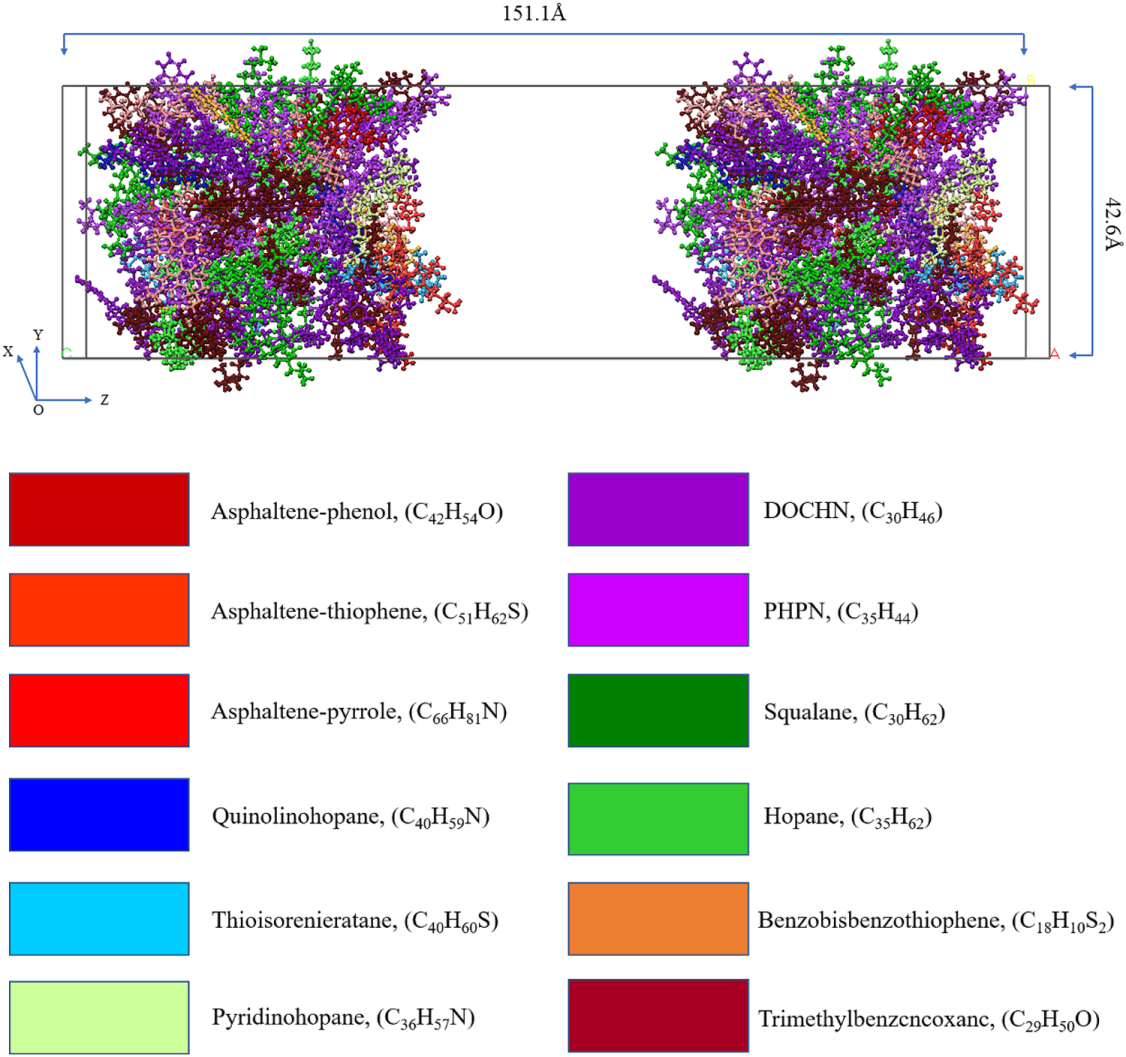
Asphalt self-healing MD model.

Time and temperature are key factors affecting asphalt self-healing^23,50^, and for electromagnetic induction heating materials, the optimal self-healing temperature is different owing to different heating times and heating process parameters^25^. Previous studies indicated that the self-healing temperature range of asphalt mixtures is between 328.15 and 363.15 K^25,51–54^; therefore, based on previous studies, 333.15 K was selected as the simulated self-healing temperature in this study, and the initial configuration of the self-healing model is shown in Fig. 4.

### Self-healing rate

In this study, the self-healing rates of several asphalts are analyzed by relative concentration and diffusion coefficient. Through the analysis of the relative concentration data, the variation rule of the crack width in the simulation is calculated. Combined with the change of OZ direction length of the system, the self-healing rate was calculated, and the calculation equations are shown in Eqs. (1)–(3)^25^.

1 $${l}_{c}=\frac{{n}_{t}}{{n}_{0}}\times {l}_{t}$$

2 $${l}_{a}={l}_{t}-{l}_{c}$$

3 $${SH}_{mic}=\frac{{l}_{v}-{l}_{c}}{{l}_{v}}\times 100\mathrm{\%}$$

where ${l}_{c}$ is the crack width, ${l}_{t}$ is the length in the OZ direction of the model at time t, ${l}_{a}$ is the width of the asphalt molecules at time t, ${l}_{v}$ is the initial crack width, ${n}_{t}$ is the number of bins with a relative concentration of less than 0.1 in the range of 200–300 bins, ${n}_{0}$ is the total number of bins, and ${SH}_{mic}$ is the asphalt self-healing rate.

The diffusion of asphalt components has been investigated from various perspectives, including the diffusion of rejuvenators^1,6,41,43,47,55–57^, diffusion between aged and fresh asphalt^58,59^, asphalt diffusion at the aggregate interface^60,61^ and diffusion at the oil–water interface^37,62–64^. It is very important to study the diffusion rule of each component to understand the asphalt self-healing mechanism. To study the diffusion rule of each component in recycled asphalt mixed with ROSL, a 10 ns MD simulation was carried out for the self-healing model, and the mean square displacement (MSD) and diffusion coefficient (D) were selected as parameters. The calculation formula is as follows:

4 $$MSD\left(t\right)={\left|{r}_{i}\left(t\right)-{r}_{i}(0)\right|}^{2}$$

5 $$D=\frac{1}{6}\underset{t\to \infty }{\mathrm{lim}}\frac{d}{dt}\sum_{i=1}^{N}\langle {\left|{r}_{i}\left(t\right)-{r}_{i}(0)\right|}^{2}\rangle$$

where MSD represents the mean square displacement of particles, ${r}_{i}(t)$ represents the coordinates of molecules at time t, ${r}_{i}(0)$ represents the coordinates at the initial time, N represents the number of diffused particles, and angle brackets represent the ensemble average.

## Self-healing behavior analysis

Taking the self-healing model of virgin asphalt as an example, the density of the initial self-healing model was only 0.484 g/cm^3^ because of the existence of the vacuum layer. As the simulation progressed, the vacuum layer gradually decreased until it disappeared, the model volume gradually decreased to a stable state, and the density of the layer model system gradually increased to a steady state; the stable density was 0.953 g/cm^3^, which is consistent with the actual situation.

### Analysis of the molecular concentration during the asphalt self-healing process

**Figure 5:**
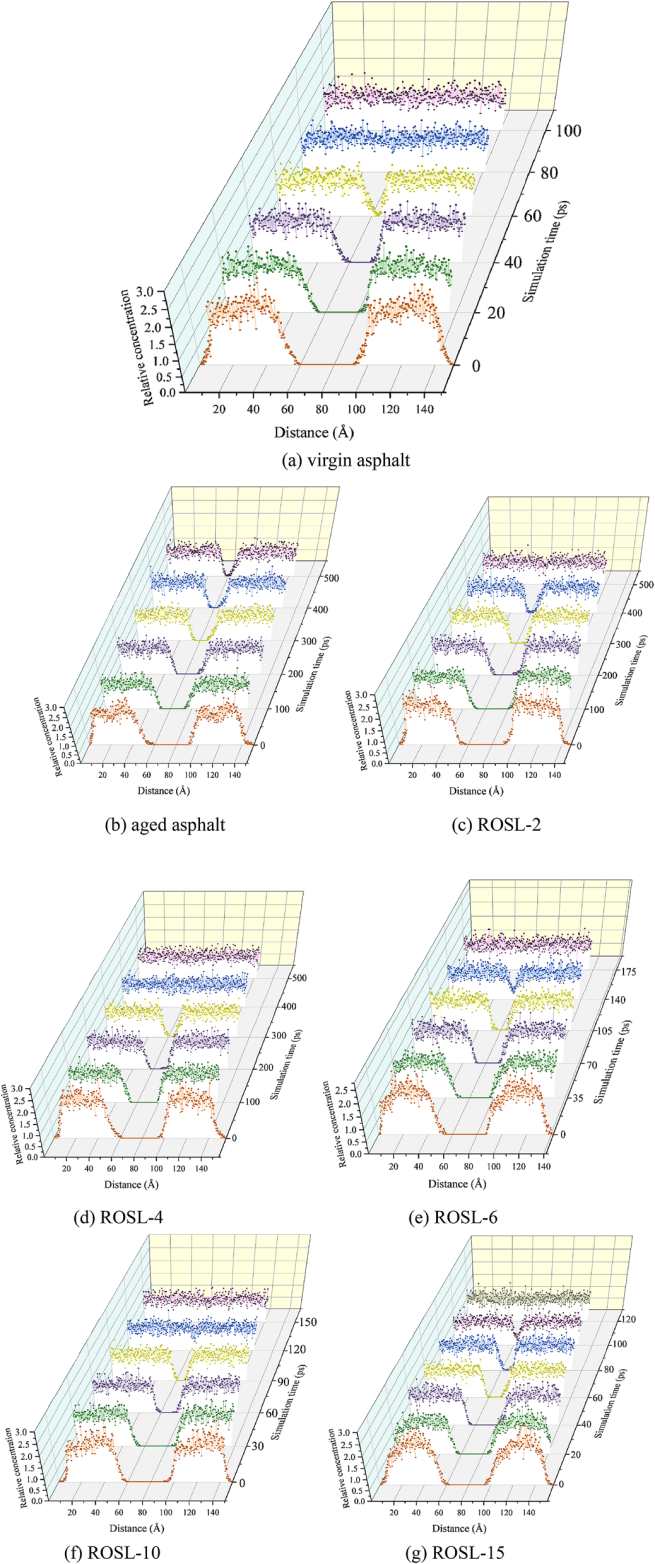
Molecular relative concentration of the asphalts MD model in the OZ direction.

**Figure 6:**
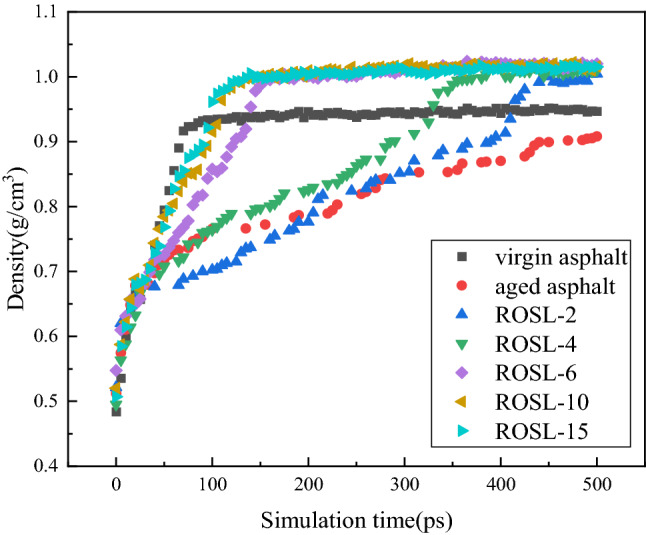
Molecular density change during self-healing process.

The asphalt self-healing model was divided into 500 bins in the OZ direction, and the relative concentration of the molecules in each bin was calculated. The model was simulated for 500 ps, and 101 conformations were generated. According to the simulation time, the conformation at the corresponding time point was selected to calculate the relative concentration. A 3D image of the relative concentration change with the simulation time is shown in Fig. 5. The change trend of the asphalt density during the self-healing process is shown in Fig. 6.

Taking the virgin asphalt in Fig. 5a as an example, the relative concentration at 0 ps presents an obvious bimodal distribution, and the peak is high and sharp. There is no molecular distribution on the boundary and middle of the periodic structure, and the range without molecular distribution in the middle is exactly the initial crack width of 30 Å. In the range of 0–60 ps, the peak relative concentration decreased significantly, indicating that the molecular concentration decreased. The width of the middle crack decreased gradually, and the molecular concentration at the edge of the model increased gradually, indicating that the asphalt molecules on both sides of the crack gradually diffused in the opposite direction and boundary during the self-healing process. At 60–80 ps, the crack disappeared, the relative concentration distribution in the entire range of the model was uniform, and the length of the model in the OZ direction was reduced to a stable state, indicating that the self-healing process had stabilized. The relative concentration distribution after 80 ps is basically consistent, indicating that asphalt self-healing enters into a slower full diffusion and integration stage.

According to the analysis shown in Fig. 5b–g, the self-healing rule of the aged asphalt and recycled asphalt is consistent with that of the virgin asphalt. However, at the end of 500 ps, the cracks inside the aged asphalt still did not disappear completely, indicating that the diffusion rate of aged asphalt molecules is slow, and it will take a longer time to heal the crack. However, with the addition of the rejuvenator, the asphalt self-healing rate was obviously accelerated, and there was a positive correlation between the self-healing rate and the rejuvenator content. The range of crack disappearance time corresponding to the rejuvenator content from 2 to 15% was 415 ps, 330 ps, 140 ps, 110 ps, and 100 ps, respectively. When the rejuvenator content was 6%, the self-healing process of recycled asphalt showed a rapid turning point and was significantly faster than that of the previous contents of 2% and 4%. When the rejuvenator contents were 10% and 15%, the self-healing process of recycled asphalt was faster than that of 6%; however, the difference was significantly smaller than the difference in the self-healing process times of the previous three types. Figure 6 also verifies the relative concentration distribution changes during the self-healing process. As seen in Fig. 6, the density order in reaching a steady state is virgin asphalt > ROSL-15 > ROSL-10 > ROSL-6 > ROSL-4, and the density of aged asphalt and ROSL-2 do not reach equilibrium, which is consistent with the order of the equilibrium times of the relative concentration.

### Stage analyses of the asphalt self-healing process

**Figure 7:**
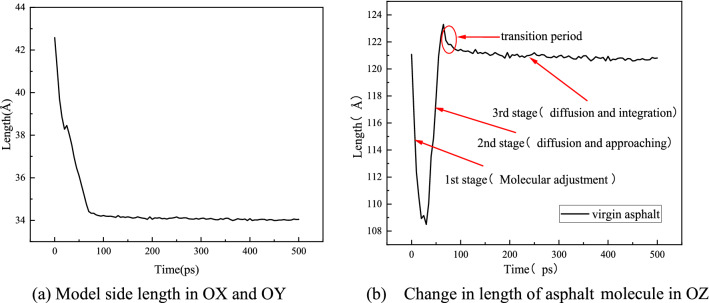
Variation in model side length and asphalt molecular length during self-healing process of virgin asphalt.

**Figure 8:**
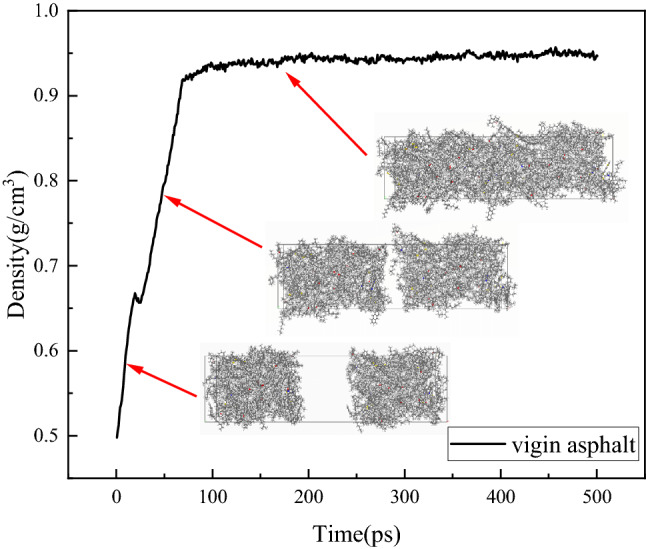
The model conformation during the self-healing process.

During the asphalt self-healing process, the change in the model side length and the length in the OZ direction of the asphalt molecules are shown in Fig. 7. In Fig. 7a, in the OX and OY directions, the side length of the virgin asphalt model decreases with time until it stabilizes. Figure 7b shows the length change in the OZ direction during the self-healing process of virgin asphalt molecules, which can be divided into three stages. The first stage occurs within 30 ps; the length of the asphalt molecules decreases with a decrease in the model volume in the early stage of self-healing. In this stage, the asphalt molecules randomly adjust and diffuse in all directions of the model, increasing the concentration of molecules at the boundary. In the second stage, within 30–65 ps, the length of the asphalt molecules gradually increases, and the molecules on both sides of the crack gradually diffuse and approach; the width of the crack gradually decreases until the molecules on both sides of the crack contact, and then the crack disappears. The third stage occurs after 65 ps, when the length of the asphalt molecules in the OZ direction is basically stable, and asphalt molecules enter a slow diffusion and integration stage. There is a transition period at the junction of the third and second stages, during which the length of the asphalt molecules decreases slightly, indicating that after the crack disappears, the asphalt molecules on both sides began to twist and integrate. Based on the above analysis, the three stages are defined as molecular adjustment, diffusion and approaching, and diffusion and integration. According to the study of Hu et al.^26,27,65,66^, the asphalt self-healing process goes through three stages: molecular approach, wetting, and diffusion, which is consistent with the above three stages in this study. Figure 8 shows the model conformation in the three stages of the self-healing process with virgin asphalt as an example.

**Figure 9:**
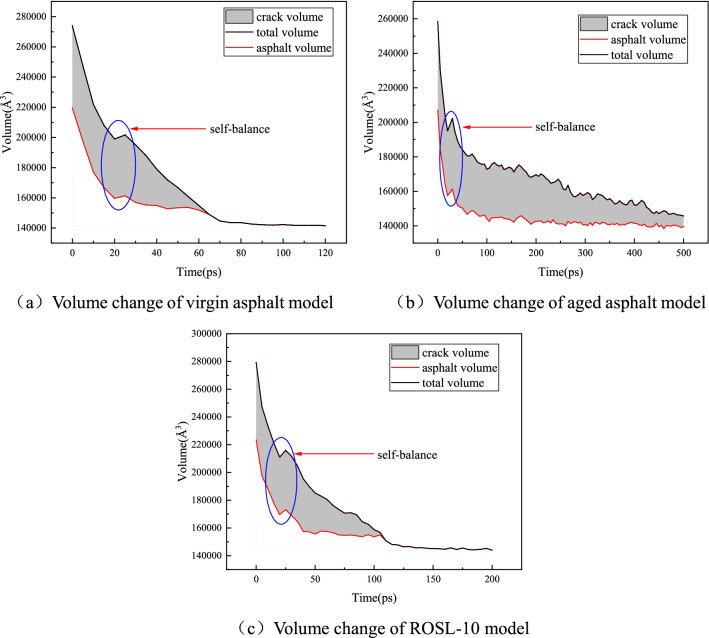
Volume change of virgin asphalt, aged asphalt and ROSL-10 models during self-healing process.

Figure 9 shows virgin asphalt, aged asphalt, and ROSL-10 as examples to calculate the total volume of the model, occupied volume of asphalt molecules, and volume of cracks. The results show that the total volume of the model decreases continuously, indicating that it is compressed, which is consistent with the change rule of the side length. The final result of compression is the complete disappearance of the crack, and the total volume of the model is the occupied volume of the asphalt molecules. It can also be seen from Fig. 9 that the asphalt volume decreases significantly in the early stage of self-healing, accompanied by a temporary equilibrium phenomenon, which lasts approximately 20–25 ps and then disappears quickly. In addition, the volume of virgin asphalt and ROSL-10 tends to be stable after 30 ps and 50 ps, respectively; however, at this time, the side lengths of the model in the OX and OY directions decrease, causing the length of asphalt molecules in the OZ direction to increase. This shows that the asphalt molecules are stretched and actively extend into the crack volume, which promotes the healing of the cracks. The compression of the model volume and stretching of the asphalt molecules are the main reasons for crack healing. For aged asphalt, after a long period of molecular movement, although the crack volume is constantly occupied by asphalt molecules, it still cannot be completely occupied; thus, it will take a longer time to heal the crack.

### Self-healing rate analysis of different asphalts

**Figure 10:**
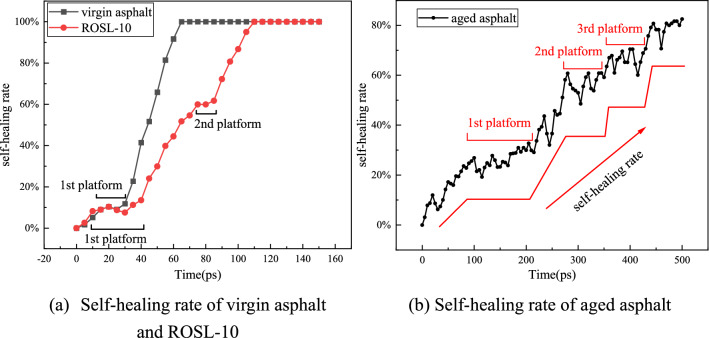
Self-healing rate of virgin asphalt, aged asphalt and ROSL-10.

Using virgin asphalt, aged asphalt, and ROSL-10 as examples, according to Eqs. (1)–(3) the self-healing rate was calculated, as shown in Fig. 10. As can be seen from Fig. 10a,b, the self-healing rate of virgin asphalt reached 100% in 65 ps, and that of ROSL-10 reached 100% in 110 ps, while the self-healing rate of aged asphalt at the end of 500 ps was only 80%. Therefore, the order of the self-healing rate of the three asphalts was virgin asphalt > ROSL-10 > aged asphalt.

During the asphalt self-healing process, different asphalts will have different “platform period” phenomena, as shown in Fig. 10. The crack width during the “platform period” remained basically unchanged and was in a dynamic equilibrium state, which is similar to the conclusions of He et al.^25^. As can be seen from Fig. 10a, for the virgin asphalt, a “platform period” only appeared within 15–30 ps during the self-healing process. This period occurred at the end of the rapid reduction of the model volume and ended at the end of the model's short-term self-equilibration. For ROSL-10, there were two “platform periods” during the self-healing process. The first one was basically the same as that of virgin asphalt, but its duration was longer, and it occurred in the range of 10–35 ps. The second “platform period” occurred within 75–85 ps. This stage was a period of self-equilibrium before crack healing in the recycled asphalt. At the end of this stage, the asphalt molecules on both sides of the crack contacted, and then healed the crack. The self-healing rate of ROSL-10 was obviously lower than that of virgin asphalt owing to the prolongation of the first “platform period” and the emergence of the second “platform period”.

As can be seen from Fig. 10b, the self-healing process of aged asphalt experienced multiple “platform periods.” Within 500 ps, there were three “platform periods.” If the simulation time is extended, the aged asphalt may experience more “platform periods” before the crack disappears. Compared with virgin and recycled asphalts, the first “platform period” of aged asphalt appeared later and lasted longer and occurred within 100–200 ps. The second “platform period” appeared within 280–345 ps, the third “platform period” platform period appeared within 365–430 ps, and their duration was longer than that of ROSL-10. During the self-healing process of aged asphalt, the healing rate developed step by step; that is, before the crack disappeared, the aged asphalt dynamically balanced once every time period. Moreover, the self-healing time of aged asphalt became longer due to the increase and prolongation of the “platform periods.” Xu et al.^40^ believed that aged asphalt has a higher activation energy barrier during the process of self-healing, which decreases its self-healing rate. Therefore, the “platform period” can be regarded as the activation energy barrier. The self-healing process of aged asphalt needs to continuously cross this barrier to make the molecules on both sides of the crack approach each other. The addition of a rejuvenator can reduce the number of “platform periods” and the activation energy barrier, which is beneficial to the self-healing ability.

### Diffusion coefficient analysis

**Figure 11:**
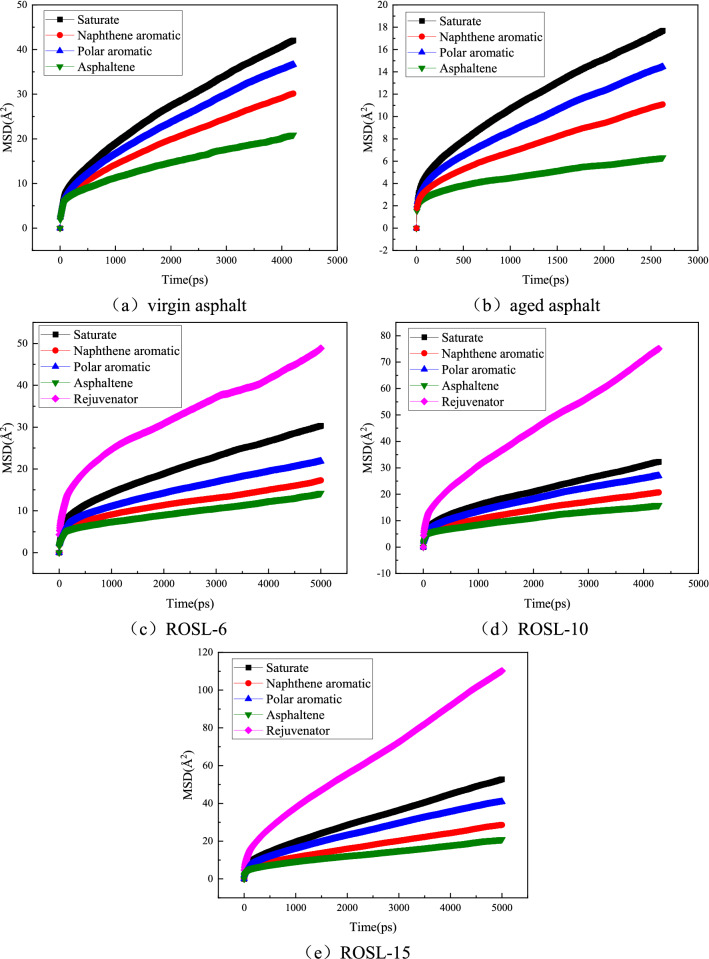
Molecular MSD of asphalt components.

**Figure 12:**
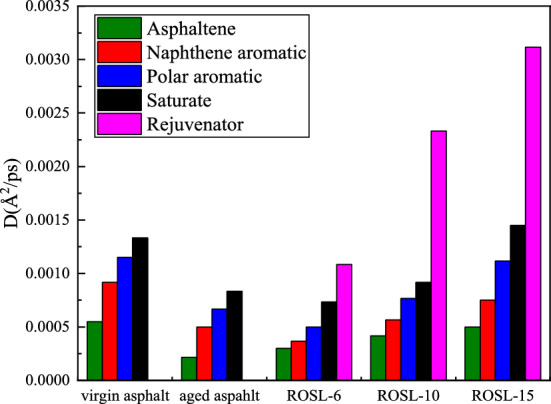
Molecular diffusion coefficient of asphalt components.

**Figure 13:**
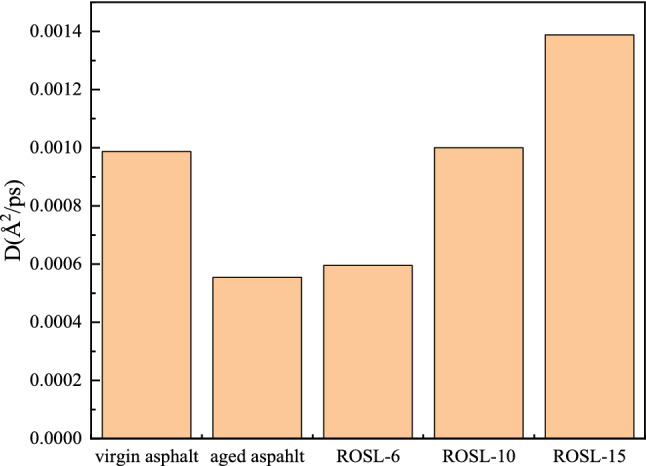
Average molecular diffusion coefficient of asphalts.

The greater the molecular diffusion coefficient is, the faster the diffusion rate is. The diffusion coefficients of virgin asphalt and aged asphalt were used as controls to observe the role of ROSL during the self-healing of recycled asphalt. According to Eqs. (4) and (5), the MSD and diffusion coefficient of each component are calculated. The results are shown in Figs. 11, 12 and 13. The recycled asphalt simulations only included ROSL-6, ROSL-10, and ROSL-15 as examples.

It can be seen from Fig. 11 that MSD has a linear relationship with time, which is in line with the view of^67^, and the diffusion coefficient D was calculated according to Eq. (5). Figure 12 shows the diffusion coefficients of the four components of asphalt and the rejuvenator during the self-healing process. It can be clearly seen in the virgin and aged asphalts that the diffusion coefficient of saturate is the largest, followed by polar and naphthene aromatics, and the diffusion coefficient of asphaltene is the smallest, indicating that saturation plays a positive role during the process of self-healing. The main component of the saturate is chain paraffins, which tend to stretch and entangle, enabling the connection of the molecules on both sides of the crack, thus promoting self-healing. The main structure of asphaltene as the core are polycyclic aromatic hydrocarbons, which have a large molar mass and high polarity. There is a strong π–π interaction between asphaltenes. Moreover, after asphalt aging, some of the polar aromatics are converted to asphaltene, which promotes the polymerization of asphaltene. As a result, the diffusion coefficient of asphaltene is the smallest. The diffusion coefficient of the rejuvenator is the largest in recycled asphalt, and the diffusion rule of the other components is the same as that of virgin asphalt. The diffusion coefficients of the four components in ROSL-6 and ROSL-10 are similar to those of the aged asphalt, indicating that the rejuvenator plays a more important role during the self-healing process. The diffusion coefficients of the four components in ROSL-15 are slightly higher than those of the other two recycled asphalts, which may be because the large content of the rejuvenator lubricates the four components, thus promoting the diffusion of the four components. The diffusion coefficient of the rejuvenator is closely related to its content, and the higher the content of the rejuvenator, the greater the diffusion coefficient.

Figure 13 shows the comparison of the average diffusion coefficients of several asphalts. The diffusion coefficient of ROSL-15 is the largest, the diffusion coefficient of ROSL-10 is basically equal to that of virgin asphalt, the diffusion coefficient of aged asphalt is the smallest, and the diffusion coefficient of ROSL-6 is slightly higher than that of aged asphalt. The results show that the self-healing rate of recycled asphalt reaches the same value as that of virgin asphalt when the content of the rejuvenator is 10%.

### Relationship between self-healing behavior and energy

**Figure 14:**
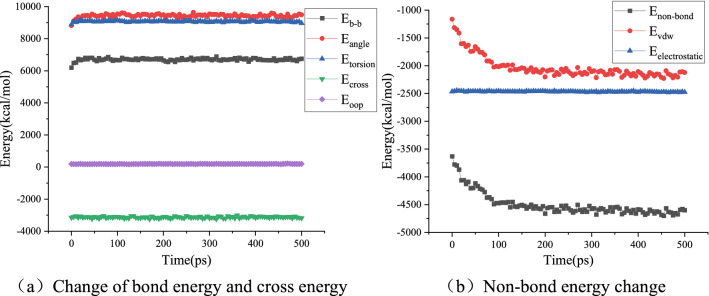
Energy change during virgin asphalt self-healing process.

**Figure 15:**
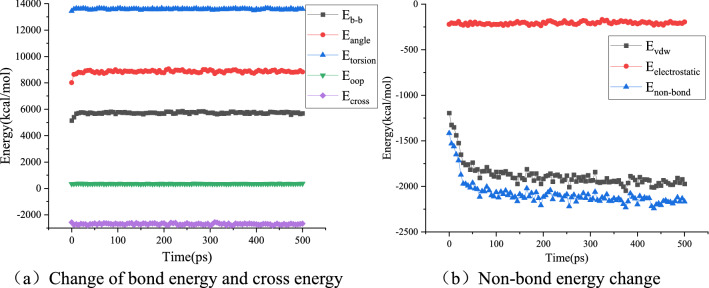
Energy change during aged asphalt self-healing process.

**Figure 16:**
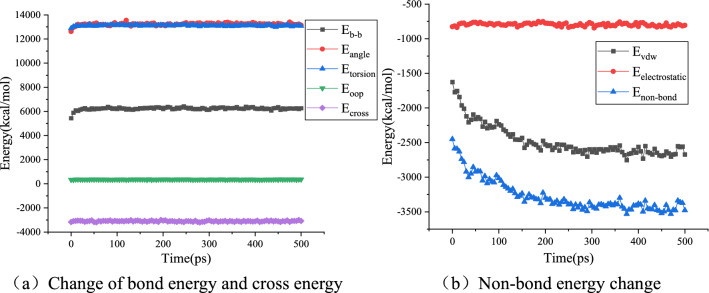
Energy change during ROSL-10 self-healing process.

The molecular force field function is composed of three parts: bond energy E_bond_, cross energy E_cross_, and non-bond energy E_non-bond_. The bond energies E_b-b_, E_angle_, E_torsion_, and E_oop_ are included in the bond energy E_bond_. The van der Waals energy E_vdw_ and electrostatic energy E_electrostatic_ belong to the non-bonding energy E_non-bond_. The calculation results for several energies are shown in Figs. 14, 15 and 16.

Taking virgin asphalt, aged asphalt, and ROSL-10 as examples, Figs. 14, 15 and 16a show that the bond energy and cross energy terms basically maintain a balance during the process of self-healing, and only fluctuate slightly at the beginning, which is a normal phenomenon. The electrostatic energy in Figs. 14, 15 and 16b does not change during the self-healing process; however, the van der Waals energy shows an obvious decreasing trend. This indicates that the asphalt phase structure evolves toward energy minimization during the self-healing process, which was confirmed by Hou et al.'s study on the asphalt self-healing mechanism that using the phase field method^20^. During the self-healing process, the order of non-bond energy is as follows: aged asphalt > ROSL-10 > virgin asphalt. When a molecule is affected by the strong gravitational force of other molecules, the movement ability is weakened, which is reflected in the weakened diffusion ability, thus affecting the asphalt self-healing rate. Aged asphalt has a large polar molecular weight and strong intermolecular force; therefore, the molecular diffusion rate is slow, and the rejuvenator can weaken the intermolecular force of the aged asphalt. Thus, the self-healing rate of ROSL-10 was higher than that of aged asphalt. Combined with the “multi-platform” phenomenon and analysis of the activation energy barrier during the self-healing process of aged asphalt in “Self-healing rate analysis of different asphalts”, the intermolecular interaction energy can be considered as the activation energy barrier. The larger the non-bond interaction energy is, the higher the activation energy barrier will be, and the more difficult it will be for the molecules to escape from the intermolecular attraction, thus decreasing the diffusion ability.

The bond and cross energies remained basically unchanged, indicating that the interatomic connections did not change significantly during the self-healing process. However, the decrease in the non-bond interaction energy indicates that the asphalt self-healing process is controlled by intermolecular forces. Because the electrostatic energy is basically unchanged, the van der Waals interaction energy decreases significantly, which indicates that the asphalt self-healing process has nothing to do with the electrostatic energy, and the van der Waals interaction energy is the main factor affecting the asphalt self-healing, which is consistent with the research conclusion of He et al.^25^.

### Analysis of the optimum rejuvenator content

**Figure 17:**
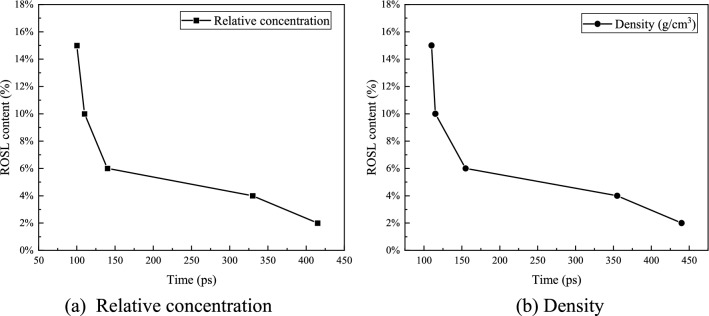
Time of relative concentration equilibrium distribution and time of density stability during the process of self-healing.

The relative concentration distribution, density change, and diffusion coefficient during the self-healing process were analyzed, as shown in Fig. 17. From 2 to 15%, the time required for the relative concentration to reach a uniform distribution decreased. The 6% content represented a turning point; compared with ROSL-4, the time for the relative concentration of ROSL-6 to reach a uniform distribution decreased from 330 to 140 ps, and the density stabilization time was shortened from 355 to 155 ps. When the content of the rejuvenator reached 10%, the time for ROSL-10 to reach the equilibrium distribution of relative concentration and the time for the density to stabilize continued to decrease; however, the time difference between ROSL-10 and ROSL-6 was smaller than that between ROSL-6 and ROSL-4. Comparing the 15% content and 10% content, it can be found that the time for the stabilization of ROSL-15 was shorter but almost insignificantly, and the time for their relative concentration distributions to reach a balanced distribution was close. In addition, when the added rejuvenator was at 10%, the diffusion coefficient of ROSL-10 was the same as that of virgin asphalt. Although the diffusion coefficient of ROSL-15 was higher than that of ROSL-10, considering that the excessive addition of rejuvenator may lead to a decline in the high-temperature performance of recycled asphalt and the occurrence of water damage^1^, it is recommended that 10% is the reasonable rejuvenator content. However, it is worth noting that there are many reasons for asphalt aging, and the asphalt will not always aged in the same way. Qu et al.^42^ proposed 12 molecular models of short-term aged asphalt and long-term aged asphalt according to the Rolling Thin Film Oven Test (RTFOT) and the Pressure Aging Vessel (PAV) test. In this paper, the study adopted his long-term aged asphalt model, 10% ROSL is suitable for this long-term aged asphalt. The recommended content can be confirmed by Ding et al.^13^. They conducted a performance test on bio-asphalt prepared using ROSL. The results showed that the performance of the bio-asphalt prepared with 10% ROSL was good. The content of the ROSL recommended in this study is consistent with their recommendations.

## Conclusions

1. The self-healing rate of virgin asphalt was the fastest and that of aged asphalt was the slowest. The self-healing rate of recycled asphalt was between these two rates, and the cracks in the aged asphalt did not disappear in the same simulation time, indicating that aging weakens the asphalt self-healing ability.
2. During the aged asphalt self-healing process, there were several “platform periods,” which lasted for a longer time. The more often the “platform period” occurred, the slower the asphalt self-healing rate was. The addition of rejuvenator can reduce the occurrence of “platform periods” and enhance the asphalt self-healing ability.
3. In the recycled asphalt, the diffusion coefficient of the rejuvenator was the largest, followed by the saturate, polar aromatic, and naphthene aromatics, and the asphaltene was the smallest. With an increase in the rejuvenator content, the average molecular diffusion coefficient of recycled asphalt gradually increased, and the average molecular diffusion coefficient of ROSL-10 was basically equal to that of virgin asphalt.
4. Adding a rejuvenator can weaken the intermolecular forces of aged asphalt and promote the diffusion ability of the molecules. Therefore, the self-healing rate of recycled asphalt is higher than that of aged asphalt. After a comprehensive analysis of the relative concentration distributions, density changes, and diffusion coefficients during the self-healing process, 10% was recommended as the optimal content of ROSL.
5. In this study, the self-healing behavior of recycled asphalt prepared by ROSL was studied at the nanoscale. Although it has not been applied in engineering, it provides good theoretical support for the reclamation of asphalt pavements in engineering. It is necessary to further analyze the microstructure and properties of recycled asphalts prepared with ROSL to establish the relationship between recycled asphalt components and service performance. The influence of temperature, crack width, and humidity on the self-healing behavior of recycled asphalt also needs to be further studied.
